# Multimodal Magnetic Resonance Imaging with Mild Repetitive Head Injury in Awake Rats: Modeling the Human Experience and Clinical Condition

**DOI:** 10.1007/s12264-025-01438-9

**Published:** 2025-06-29

**Authors:** Nicole Bens, Arnold Chang, Richard Ortiz, Joshua Leaston, Praveen Kulkarni, Rosemarie Hightower, Sophia Prom, Nicholas O’Hare, Eno Ebong, Craig F. Ferris

**Affiliations:** 1 Center for Translational Neuroimaging, Northeastern University, Boston, MA 02115 USA; 2 Department of Psychology, Northern Illinois Univ, DeKalb, IL 60112 USA; 3 Stanford Medical School, Palo Alto, CA 94301 USA; 4 Department of Bioengineering, Northeastern University, Boston, MA 02115 USA; 5 Department of Chemical Engineering, Northeastern University, Boston, MA 02115 USA; 6 Departments of Psychology and Pharmaceutical Sciences, Northeastern University, Boston, MA 02115 USA

**Keywords:** Astrogliosis, Cerebral small vessel disease, Concussion, Ferumoxytol

## Abstract

Mild repetitive head injury is a serious health problem with long-term negative consequences. Changes in brain neurobiology were assessed with MRI in a model of head injury designed to reflect the human experience. Rats were maintained on a reverse light-dark cycle and head impacted daily at 24 h intervals over three days while fully awake under red light illumination. There was no neuroradiological evidence of brain damage. Rats were imaged for changes in blood brain barrier permeability, edema and gray matter microarchitecture, and resting state functional connectivity. Data were registered to a 3D MRI rat atlas with 173 segmented brain areas providing site-specific information on each imaging modality. Changes in BBB permeability were minimal and localized to the hippocampus and cerebellum. There was evidence of cytotoxic edema in the basal ganglia, thalamus, and cerebellum. There was a global decrease in connectivity and an increase in gliosis in the thalamus, cerebellum, and hippocampus. This study shows a sequelae of neuropathology caused by mild repetitive head injury that is commonly observed in clinical practice using MRI in patients. As such, it may serve as a model for testing the efficacy of new therapeutics using any or all of the measures as biomarkers to assess drug efficacy.

**Supplementary Information:**

The online version contains supplementary material available at 10.1007/s12264-025-01438-9.

## Introduction

According to the Centers for Disease Control and Prevention, approximately 2.9 million people in the United States experience traumatic brain injuries annually [1]. The body of research on the behavioral and neurobiological effects of repetitive mild head injuries sustained during organized sports, car accidents, falls, or military service is growing. In fact, these mild head impacts represent more than 75% of all brain injuries [2]. The diagnostic guidelines for mild head injuries, as provided by the Centers for Disease Control and Prevention, World Health Organization, and American Congress of Rehabilitation Medicine, include self-reported symptoms such as brief confusion, disorientation, impaired consciousness, or memory problems at the time of injury, without any neuroradiological signs of structural brain damage. [2, 3]. The impact of a single mild head injury is challenging to evaluate, as any neurobiological, cognitive, or behavioral issues may resolve within hours. However, a more concerning issue emerges with repeated mild head injuries. Repeated mild traumatic brain injury (rmTBI) is linked to more serious and prolonged cognitive, motor, and behavioral difficulties, which can persist for months or even years. [4, 5]. Even after the remission of symptoms, there is accumulating evidence that mild traumatic brain injuries carry an increased risk of Alzheimer’s disease [6, 7] and Parkinson’s disease [8, 9]

There are hundreds of published studies in animals modeling rmTBI using different parameters. Most have been done using a weight drop method in male mice and rats under anesthesia and during the light phase of the L-D cycle when rodents are normally sleeping [10]. A select few have avoided the confound of anesthesia [11–13]. There are wide variations in the number of impacts given in a single day and the number of days rodents are subjected to insult. For example, mice have been given as many as six mild impacts a day for seven consecutive days [11], one a day for five consecutive days [14], and as few as one hit for two consecutive days [15]. In this study a closed-head, momentum exchange model was chosen to produce mild head impacts that cause no brain damage or contusion [16, 17]. Diffusion-weighted imaging (DWI) with measures of apparent diffusion coefficient (ADC) and fractional anisotropy (FA) was used as a surrogate marker for edema and gray matter microarchitecture [18]. A newly developed imaging modality, quantitative ultrashort time-to-echo contrast enhanced (QUTE-CE) was used to assess changes in BBB permeability at the level of the microcirculation [19]. To make the studies more relevant to the human experience, rats were impacted while fully awake and during the dark phase of their L-D cycle when they are normally active. Rats were impacted once daily for three consecutive days, an occurrence that seems reasonable in organized sports. To the best of our knowledge, this model is unique to the field of preclinical head injury. Our previous publication using three mild head impacts, one a day for three consecutive days was performed in male rats, under isoflurane anesthesia and during the light phase of the L-D cycle [17]. Three weeks post head injury rats were imaged for changes in functional connectivity and analyzed for postmortem gliosis. Here, we repeated this study using females, impacted fully awake with no anesthesia, and during the dark phase of the L-D cycle. The data from each of these different head injury studies are compared. The significance of these data and their relevance to the human condition as reflected by clinical rmTBI data obtained using multimodal MRI are discussed.

## Methods

### Animals

Fourteen female Sprague Dawley rats, approximately 100 days old and weighing between 250-275g, were obtained from Charles River Laboratories (Wilmington, MA, USA). The rats were kept on a reverse 12:12 light-dark cycle (lights off at 9:00 h), in a controlled environment with an ambient temperature of 22–24°C, and had unrestricted access to food and water. All experiments were performed under dim red light between 10:00 h and 18:00 h to avoid the light-dark cycle transitions. Using the means and SDs from previous studies from our lab [16–18, 20] we calculated an estimated minimum sample size of six subjects for each experimental group using a two-tailed test, with an alpha of 0.05, beta of 0.10, and power of 0.90. The fourteen rats were divided into two separate studies. Rats were randomly assigned to two experimental groups: 1) sham controls with no head impacts (*n =* 6) and 2) head impacted (*n =* 8). Rats from this study were analyzed for changes in gray matter microarchitecture, small vessel permeability, and resting state connectivity. All animals were cared for in accordance with the NIH Guide to the Care and Use of Laboratory Animals. Methods and procedures used in this study were pre-approved by the Northeastern University Institutional Animal Care and Use Committee, protocol # 21–0824. The protocols used in this study followed the ARRIVE guidelines for reporting *in vivo* experiments in animal research [21]. Throughout the study, the animals were monitored daily for overall health, as well as their food and water intake. A 15% reduction in body weight was established as the humane endpoint for intervention.

### Head Impacts

**Fig. 1:**
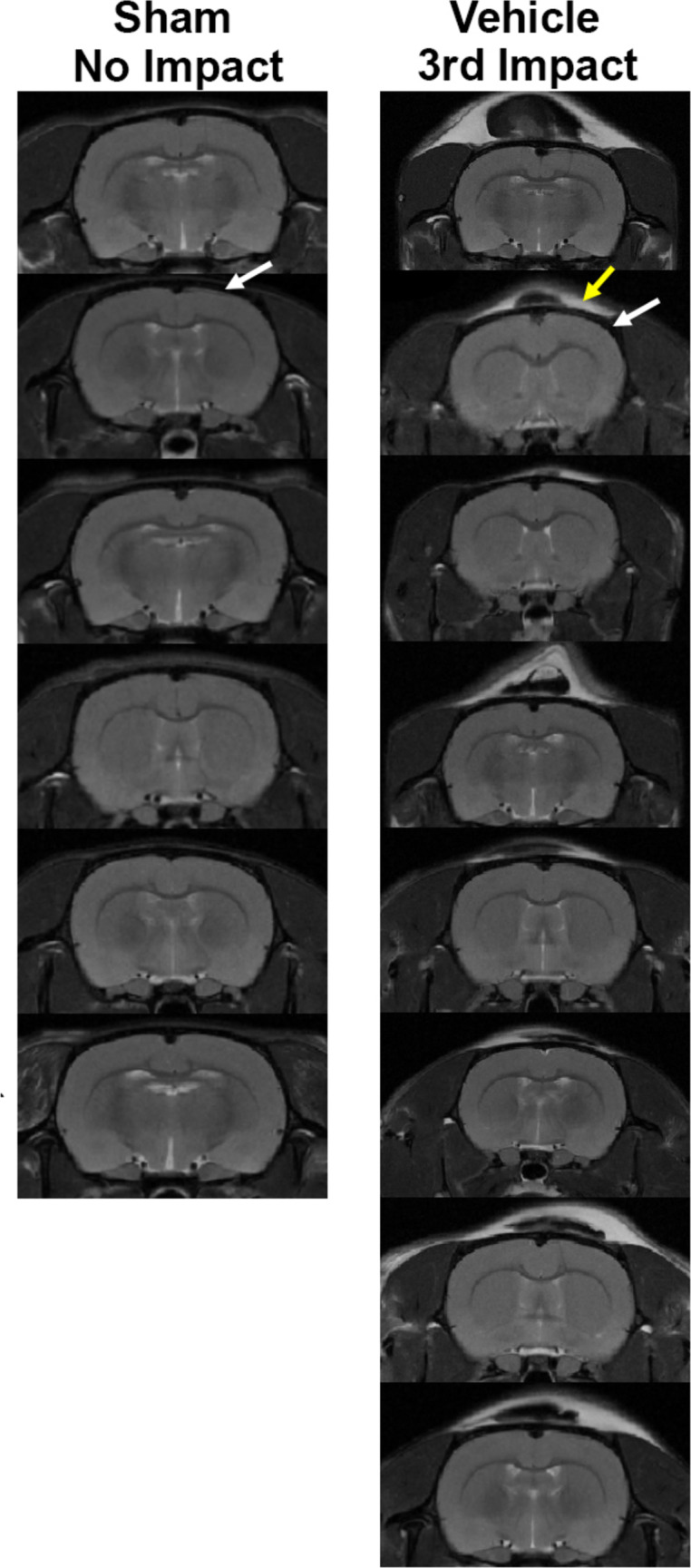
Brain scans following head impact. Shown are MRI images of frontal sections at the level of head impact for all no-hit (*n =* 6) and head-injured rats (*n =* 8). Data were collected at the end of three sham or three head impacts each separated by 24 h. The white arrows denote the skull (black) overlaying the brain while the yellow arrows show the edema (white) associated with the skin at the point of head impact. Note the absence of skull damage or any contusion in the underlying gray matter of the cortex.

Head impacts were delivered using a pneumatic pressure drive with a 50 g compactor, consistently generating impact velocities of 7.4 m/s to induce mild head injury in rats. The kinetic energy at impact was approximately 1.37 joules. This model has been previously used to study the neuroradiological effects of repetitive mild head injury in rats [16, 17]. It is comparable to the CHIMERA model used for mild head injury in mice [22, 23], with the key difference being that the rats were impacted while fully awake and during their active phase of the light-dark cycle. All rats displayed normal walking behavior within seconds of being returned to their home cages after the impact, with no mortalities or signs of skull damage or contusion observed (Fig 1).

Sham and head-impacted rats were pretreated with one dose of slow-release buprenorphine (0.1 mg/kg IP) 10 min prior to the first head impact to minimize the pain over the three-day protocol. Rats were lightly anesthetized with 2% isoflurane. A mark was placed on the skin at the top of the skull, midline at the level of the ears, approximating Bregma (See Supplementary Fig S1 for impact site). The head and body were mounted on a sled which provided complete movement along the rostral/caudal axis and rotation in the x-y plane (see Supplementary Fig S1). The impact piston and the marking on the head were aligned. When the rat was awake (ca 2–3 min) as judged by eye blink and response to toe pinch, it was impacted. Rats were subjected to three mild head impacts separated by 24 h each as previously described [17]. Neuroradiological evidence of the edema on the skin of the skull at the point of impact is shown in Fig 1. All rats were imaged for edema and changes in gray matter microarchitecture using DWI within one hour of the third head impact. On the following day, rats were imaged for BBB permeability and tested for mobility and anxiety. Two days post-final head impact rats were tested for cognition. Two weeks later rats were imaged for functional connectivity. Afterward, they were euthanized, and the brain was harvested for histology. Previous studies on repeated mild head injuries from our lab have waited between three to nine weeks before assessing postmortem pathology [17, 24]. A timeline of the experimental protocol is provided in Fig S1.

## Behavior

### Open Field

Testing in the open field was used to assess anxiety, exploratory behaviors, and locomotion [25]. Animals were placed in a large open top, black Plexiglas box and allowed to explore for 20 minutes. The box was dimly illuminated with two 40 W incandescent red-light bulbs. For analysis, the arena was divided into a peripheral zone measuring 8 cm from the edge of the arena walls with the remainder of the field (40%) defined as the central zone. The amount of time spent in the periphery and center and the total distance traveled were determined using ANY-MAZE tracking software. Each measure for the two experimental groups was compared with a t-test using GraphPad Prism version 9.1.2 for Windows, (GraphPad Software, San Diego, California USA).

### Novel Object Recognition

A novel object recognition test (NOR) was used to assess episodic learning and memory related to stimulus recognition [26]. All studies were performed on a reverse L-D cycle under dim red illumination. The apparatus consisted of a large black Plexiglas box (L:60.9 W: 69.2 H:70.5 cm) with no top. The task was performed over the course of two days starting 48 h after the third head impact. Animals were placed in an empty box (15 min) for acclimation on day one. On day two, for the familiar phase (5 min), animals were placed in a box with two identical objects arranged in diagonal corners, 5 cm from each wall. After a 90 min rest period in their home cage, animals were placed back in the box for the novel phase (3 min) with one of the familiar objects and a novel object.

The rats were video recorded and analyzed using manual methods by experimenters who were blind to treatment conditions and verified with automated scoring using ANY-maze® software (Stoelting, Wood Dale, IL, USA). Investigation ratios (IR = time spent investigating the novel object/time spent investigating both objects) were assessed using single-sample, two-tailed t-tests, and performance was compared to chance (i.e., IR = 0.5). An investigation ratio significantly greater than 0.5 indicates that the mice were spending more time with the novel object. Conversely, a ratio significantly smaller than chance indicates a preference for the familiar object. Analysis was performed with GraphPad Prism.

### Imaging

 Each day of imaging had a mix of sham and impacted rats known by all the investigators. Imaging sessions were performed using a Bruker Biospec 7.0 T/20-cm USR horizontal magnet (Bruker, Billerica, MA, USA) with a 2T/m magnetic field gradient insert (ID = 12 cm) capable of a 120-μs rise time. Radiofrequency signals were transmitted and received via a quadrature volume coil integrated into the rat restrainer (Ekam Imaging, Boston, MA, USA). The restraining system was designed with a padded head support, eliminating the need for ear bars and reducing discomfort while minimizing motion artifacts. All rats were imaged under 1%–2% isoflurane anesthesia, maintaining a respiratory rate of 40–50 breaths per minute. At the start of each imaging session, a high-resolution anatomical dataset was obtained to evaluate structural damage using the rapid acquisition, relaxation enhanced (RARE) pulse sequence (RARE factor 8) with the following parameters, 35 slices of 0.7mm thickness; field of view [FOV] 3 cm; 256 × 256; repetition time [TR] 3900 ms; effective echo time [TE] 48 ms; number of average excitations (NEX) 3; 6 min 14 s acquisition time.

#### Diffusion Weighted Imaging–Quantitative Anisotropy

DWI was acquired with a spin-echo echo-planar-imaging (EPI) pulse sequence having the following parameters: TR/TE=500/20 ms, eight EPI segments, and 10 non-collinear gradient directions with a single b value shell at 1000 s/mm^2^ and one image with a B-value of 0 s/mm^2^ (referred to as B0). Geometrical parameters were: 48 coronal slices, each 0.313 mm thick (brain volume) and with an in-plane resolution of 0.313 × 0.313 mm^2^ (matrix size 96 × 96; FOV 30 mm^2^). The imaging protocol was repeated two times for signal averaging. Each DWI acquisition took 35 min and the entire MRI protocol lasted ca. 70 min. Image analysis included DWI analysis of the DW-3D-EPI images to produce the maps of apparent diffusion coefficient (ADC) and fractional anisotropy (FA). DWI analysis was implemented with MATLAB and MedINRIA (1.9.0; http://www-sop.inria.fr/asclepios/software/MedINRIA/index.php) software. Because sporadic excessive breathing during DWI acquisition can lead to significant image motion artifacts that are apparent only in the slices sampled when motion occurred, each image (for each slice and each gradient direction) was screened, prior to DWI analysis, for motion artifacts; if found, acquisition points with motion artifacts were eliminated from analysis. For statistical comparisons among rats, each brain volume was registered to the 3D MRI rat brain atlas for the generation of voxel- and region-based statistics. All image transformations and statistical analyses were carried out using the in-house EVA software (Ekam Solutions LLC, Boston, MA, USA). Statistical differences in measures of DWI between experimental groups were determined using a nonparametric Kruskal Wallis multiple comparisons test (critical value set at <0.05) followed by post hoc analyses using a Wilcoxon rank-sum test for individual differences. The organization of brain areas into brain regions for the final analysis of ADC and FA values is provided in Supplementary Data Excel S1 & S2.

#### Blood Brain Barrier Permeability-QUTE-CE Imaging

Rats were imaged prior to and following an i.v. bolus of 6 mg/mL Fe of ferumoxytol. The injected volume was tailored for each rat (assuming 7% blood by body weight) to produce a starting blood concentration of 200 μg/mL Fe. 3D-QUTE image acquisition parameters were TE=13 µs, TR=4 ms, and flip angle (FA) = 20° with a RF hard pulse bandwidth of 200kHz. Therefore, the pulse duration was short (6.4 µs) compared to the T2 of the approximate ferumoxytol concentration (4.4ms for 3.58mmol/L, i.e. 200 µg/mL) to minimize signal blur and reduce the probability for a curved trajectory of the magnetization vector Mz. A 3 × 3 × 3 cm^3^ FOV was used with a matrix size of 180 × 180 × 180 to produce 167 µm isotropic resolution.

To ensure accurate region-based analysis, a regional atlas was developed from the 3D Rat MRI atlas in collaboration with Ekam Imaging. Each rat data set was individually adjusted to account for differences in brain size and orientation. After co-registering the images to this atlas, we applied custom MATLAB code to isolate specific brain regions for quantitative CBV (qCBV) analysis. The intensity data were extracted voxel by voxel for each region, and both mean and median intensities were computed using standard MATLAB functions. The difference between these values was then used to assess the skewness of the regional intensity distributions, providing additional insights into data variability. The equation for qCBV was as follows qCBV=(I’M-IM)/(I’B-IB), where prime donates intensity after injection, IM is the measured voxel intensity, and IB is the measured blood intensity. To determine if changes in BBB permeability could be detected at the individual animal level, one-tailed linear regression was utilized to evaluate increases in permeability over time and was followed by subsequent multiple comparisons correction. In this design, BBB permeability was quantified by analyzing the slope of the CBV vs. time curve over consecutive post-contrast scans. Fluctuations in CBV in each region of interest were attributed to modulations in BBB permeability and were calculated by the change in apparent CBV per second.

### Image Processing

Images were subjected to both motion correction and spatial realignment and then resliced with nearest neighbor algorithms with MATLAB SPM12 toolbox. The 3D MRI rat brain atlas was registered for each subject to segment brain regions for region-of-interest selection. MATLAB was utilized for all additional atlas segmentation adjustments. A homogenous copper sulfate 10-mL tube phantom was utilized to correct the image intensity for B1 coil sensitivity along the Z axis. An anatomical description of atlas regions by cluster can be found in Supplementary Table S3.

### Statistical analysis for QUTE-CE

Group analyses were conducted with t-tests to assess the effect of mild head impacts on each brain region. To control for the effect of both type-I error and type-II error, multiple comparisons correction was applied through the two-stage Benjamini, Krieger, and Yekutieli FDR method with a false discovery rate (*P<*0.05 & FDR=0.1) for each family of hypotheses. To determine if changes in BBB permeability could be detected at the individual animal level, one-tailed linear regression was utilized to evaluate increases in permeability over time and was followed by subsequent multiple comparisons correction. All analyses were performed in MATLAB, SPSS Version 27, and GraphPad Prism.

### Resting State Functional Connectivity

#### Image Acquisition

Scans were acquired using a spin-echo triple-shot EPI sequence with the following imaging parameters: matrix size = 96 × 96 × 20, TR/TE = 1000/15 ms, voxel size = 0.312 × 0.312 × 1.2 mm^3^, slice thickness = 1.2 mm, with 200 repetitions, and a total acquisition time of 15 minutes. For preprocessing, we used a range of software tools, including Analysis of Functional NeuroImages (AFNI_17.1.12), the FMRIB Software Library (FSL, v5.0.9), Deformable Registration via Attribute Matching and Mutual-Saliency Weighting (DRAMMS 1.4.1), and MATLAB. Brain tissue masks for resting-state functional images were manually created with 3DSlicer and used for skull-stripping. Motion outliers, or data segments affected by significant movement, were identified and recorded for later regression, and large motion spikes were removed from the time-course signals. Slice timing correction was applied to adjust for interleaved slice acquisition order. Head motion correction was performed using the six motion parameters, with the first volume as the reference image. Functional data were normalized to the 3D MRI Rat Brain Atlas © via affine registration using DRAMMS, which includes 173 annotated brain regions for segmentation. After quality control, a band-pass filter (0.01 Hz to 0.1 Hz) was applied to minimize low-frequency drift and high-frequency physiological noise. The images were then detrended and spatially smoothed with a full width at half maximum of 0.8 mm. Additionally, regressors for motion outliers, the six motion parameters, mean white matter, and cerebrospinal fluid time series were included in general linear models for nuisance regression to remove unwanted effects.

To assess the correlations in spontaneous BOLD fluctuations, a region-to-region functional connectivity analysis was performed. This analysis involves a network made up of nodes (regions of interest or ROIs) and edges (connections between these regions). We averaged the voxel time series data within each node based on residual images from the nuisance regression process. Pearson’s correlation coefficients were calculated for all possible pairs of nodes (14,535 pairs) for each subject across all three groups to evaluate interregional temporal correlations. The resulting *r*-values, which range from −1 to 1, were transformed into Z-scores using Fisher's Z transformation to improve normality. We created symmetric connectivity matrices of size 166 × 166, where each entry reflects the strength of an edge. A group-level analysis was then performed to investigate functional connectivity within the experimental groups. The Z-score matrices from one-group *t*-tests were clustered using the K-nearest neighbors'method to determine how nodes group together to form resting-state networks. To enhance visualization, a Z-score threshold of |Z| = 2.3 was applied to remove weak or spurious node connections.

### Functional Connectivity Analysis

#### Degree Centrality

We performed all network analysis using Gephi, an open-source tool for visualizing and analyzing networks [27]. We imported the absolute values of the symmetric connectivity matrices for both experimental groups, treating the edges as undirected networks. Degree centrality analysis measures the number of connections that a particular node has within the entire network. Degree centrality is defined as:

$${\text{C}}_{{\text{D}}} \left( {\text{j}} \right) = \mathop \sum \limits_{{{\text{j}} = 1}}^{{\text{n}}} {\text{A}}_{{{\text{ij}}}}$$

In this context,"n"denotes the total number of rows in the adjacency matrix labeled"A,"and the elements within the matrix are represented as"Aij,"indicating the number of edges between nodes i and j.

### Statistics

We carried out all statistical analyses for the graph theory assessment using GraphPad Prism. To determine whether parametric or non-parametric methods were suitable for different group subregions, we first performed normality tests using Shapiro-Wilk's test. Subregion degree centrality p-values greater than 0.05 indicated a normal distribution. After confirming normality, we used paired *t*-tests to compare the degree of centrality between experimental groups in various subregions. When normality could not be assumed, we applied the nonparametric Wilcoxon signed-rank (WSR) test.

### Immunohistochemistry

Eighteen days after the third head impact rats from the sham group (*n =* 5), the head impacted group (*n =* 4), were anesthetized with 3% isoflurane and transcardially perfused with PBS followed by 4% paraformaldehyde (PFA) solution. The timing of the tissue collection was designed to coincide with a recent study showing changes in gliosis after repetitive mild head injury [24]. After brains were removed, they were further fixed in 4% PFA for 24 h and subsequently preserved in 30% sucrose in PBS at 4°C. Brain tissues, frozen on OCT and stored at 80 °C, were then sectioned using a cryostat with a thickness of 50 microns. The sectioning was done in sequential order across 5 wells with 250 µm intervals between each section in each well. Sections were placed in PBS and used one day later for immunostaining. The steps for immunohistochemistry and analysis have been published [20, 24].

## Results

### Behavior Tests

**Fig. 2:**
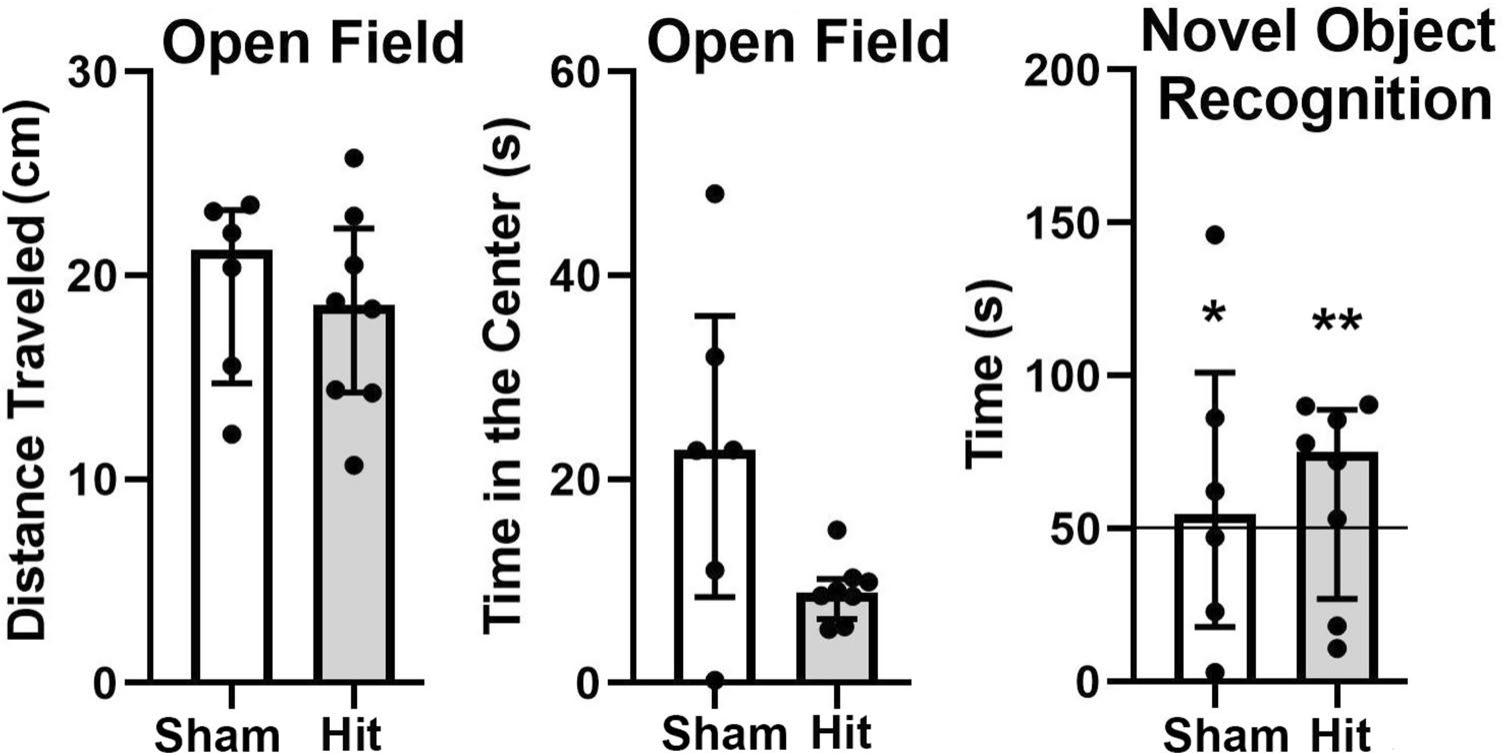
Open Field and Novel Object Recognition. Scatter plots with the median and interquartile range are shown for each behavioral test. The experimental groups include sham controls without head injury (*n =* 6) and rats hit three times. Left and Center: There were no significant differences in distance traveled or time spent in the center between groups in the Open Field. Right: In the NOR test, there were no significant differences in time spent investigating the novel object among the groups. However, both groups were significantly greater than chance (50%) investigating the novel object. * P <0.05; ** *P* <0.01.

Data from the Open Field test and NOR are shown in Fig. 2. The bar graphs show the inter-quartile range and value for each subject for no-hit controls (Sham) and female rats hit three times. There were no significant differences between groups for distance traveled or time spent in the center zone. While there were no significant differences between groups for NOR, each group was significantly greater than chance (50%) when exploring the novel object.

### Diffusion-Weighted Imaging

**Fig. 3:**
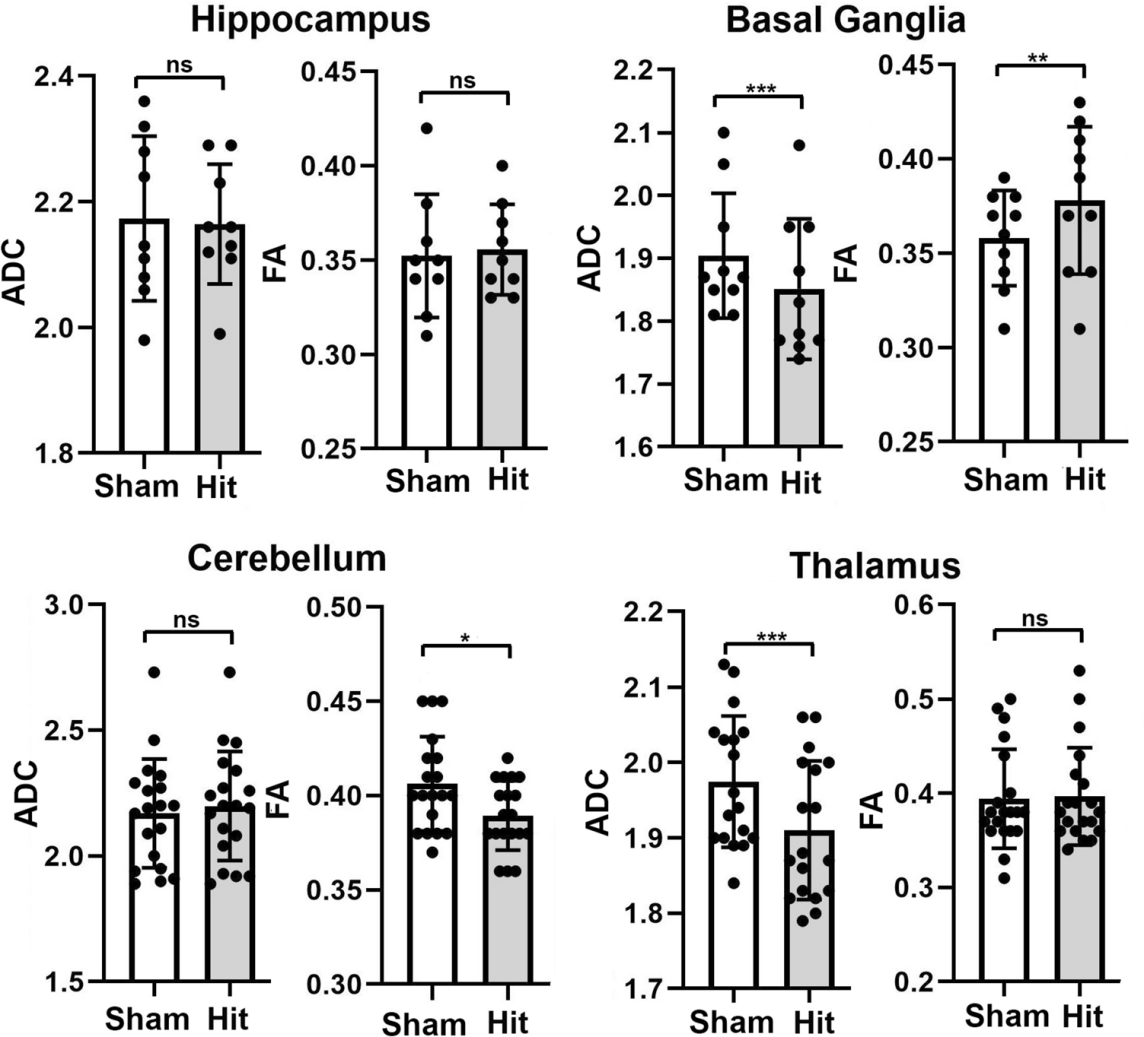
Diffusion Weighted Imaging. Dot plots with the mean ± SD are shown for two indices of anisotropy – apparent diffusion coefficient (ADC) and fractional anisotropy (FA). The dots represent the number of brain areas in that particular brain region. For example, the hippocampus comprised of the dorsal and ventral dentate, dorsal, and ventral subiculum, dorsal and ventral CA1, dorsal and ventral CA3, and CA2 has nine dots. Each dot is the average of the values for that particular brain area in sham controls without head injury (*n =* 6), and rats hit three times. The list of brain areas that comprise basal ganglia, hippocampus, cerebellum, and thalamus can be found in Supplementary Data File S3. *P<0.05; ** P <0.01; *** P <0.001.

Shown in Fig. 3 are bar graphs (mean ± SD) and dot plots (brain areas) for each subject for ADC and FA values in different brain regions for sham and hit rats. For the hippocampus, comprised of nine brain areas, there were no significant differences between ADC and FA as compared to sham-treated rats. In the basal ganglia, comprised of ten brain areas, there was a significant decrease in ADC in hit rats as compared to sham (*P<*0.001) and an increase in FA values (*P<*0.01). The cerebellum, comprised of nineteen brain areas, shows a significant reduction in FA in rats hit as compared to shams (*P<*0.05). The thalamus, comprised of eighteen brain areas, showed significantly lower measures of ADC (*P<*0.001) as compared to sham.

### Blood Brain Barrier Permeability

**Fig. 4.**
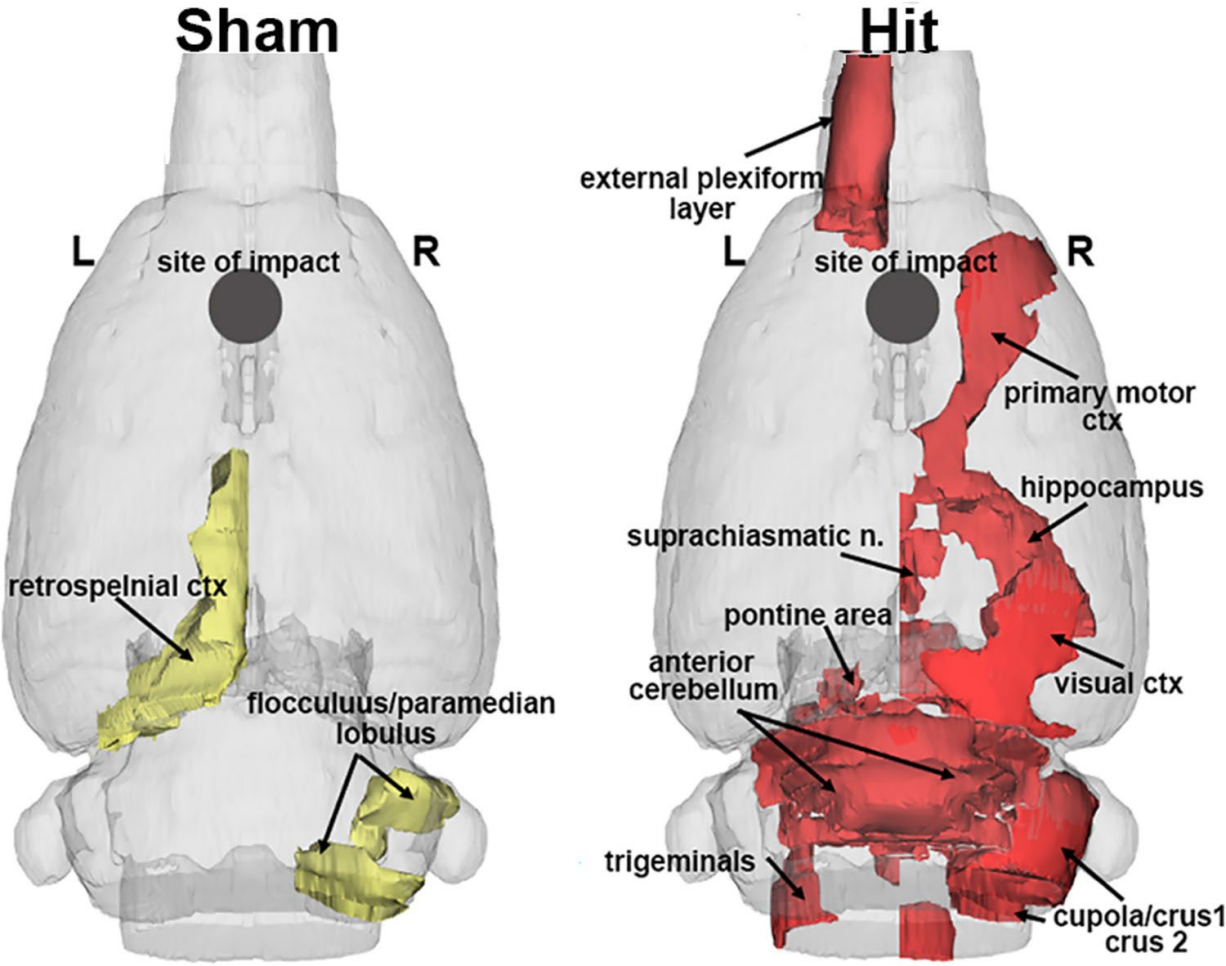
BBB Permeability. Shown are color-coded, 3D reconstructions of brain areas with significant changes in blood brain barrier permeability for both experimental conditions in response to head impact in the area of the forebrain (black dot). These single brain areas were lateralized to either the left or right sides

The changes in BBB permeability measured 24 hrs after the third head impact were minimal. When the brain is organized into 116 bisymmetrical regions (58 left and 58 right, see Supplementary File S3), two of the eight sham controls presented with significant permeability in just two areas, the retrosplenial ctx and flocculus/paramedian lobule of the cerebellum, shown highlighted in yellow in Fig. 4. Four of the seven head-impacted rats presented with significant permeability in 12 brain regions shown highlighted in red in Fig. 4. These areas include the hippocampus, brainstem, and cerebellum.

### Functional Connectivity

**Fig. 5.**
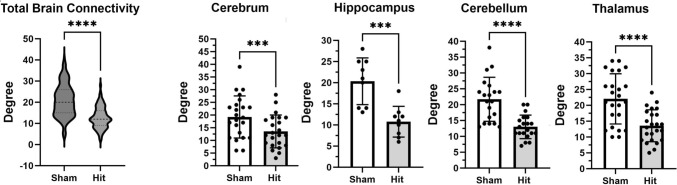
Functional Connectivity. The degrees (connections) for all 173 brain areas between sham and impacted rats are shown as violin plots. There is a significant decrease in global connectivity with head injury (two-tailed, *t*-test, *P<*0.0001). When the 173 brain areas were organized into 12 brain regions there were four brain regions that showed significant differences in connectivity. These differences are presented in dot plots and bar graphs (mean ± SD) for the cerebrum, hippocampus, cerebellum, and thalamus. The dots are the individual brain areas that make up the brain regions. *** *P<*0.001; **** *P<*0.0001.

Shown in Fig. 5 is a violin plot of all 173 brain areas representing total brain connectivity. Rats impacted three times have significantly (*P<*0.0001) fewer connections (degree) than sham controls. The decrease in connectivity for specific brain areas is shown in the dot plots (mean ± SD) to the right. Global network analysis shows an Average Degree of 20.83 vs 12.78, Graph Density of 0.123 vs 0.075, and Average Path Length of 2.183 vs 2.507 for head impacted and shams, respectively.

### Histology

**Fig. 6.**
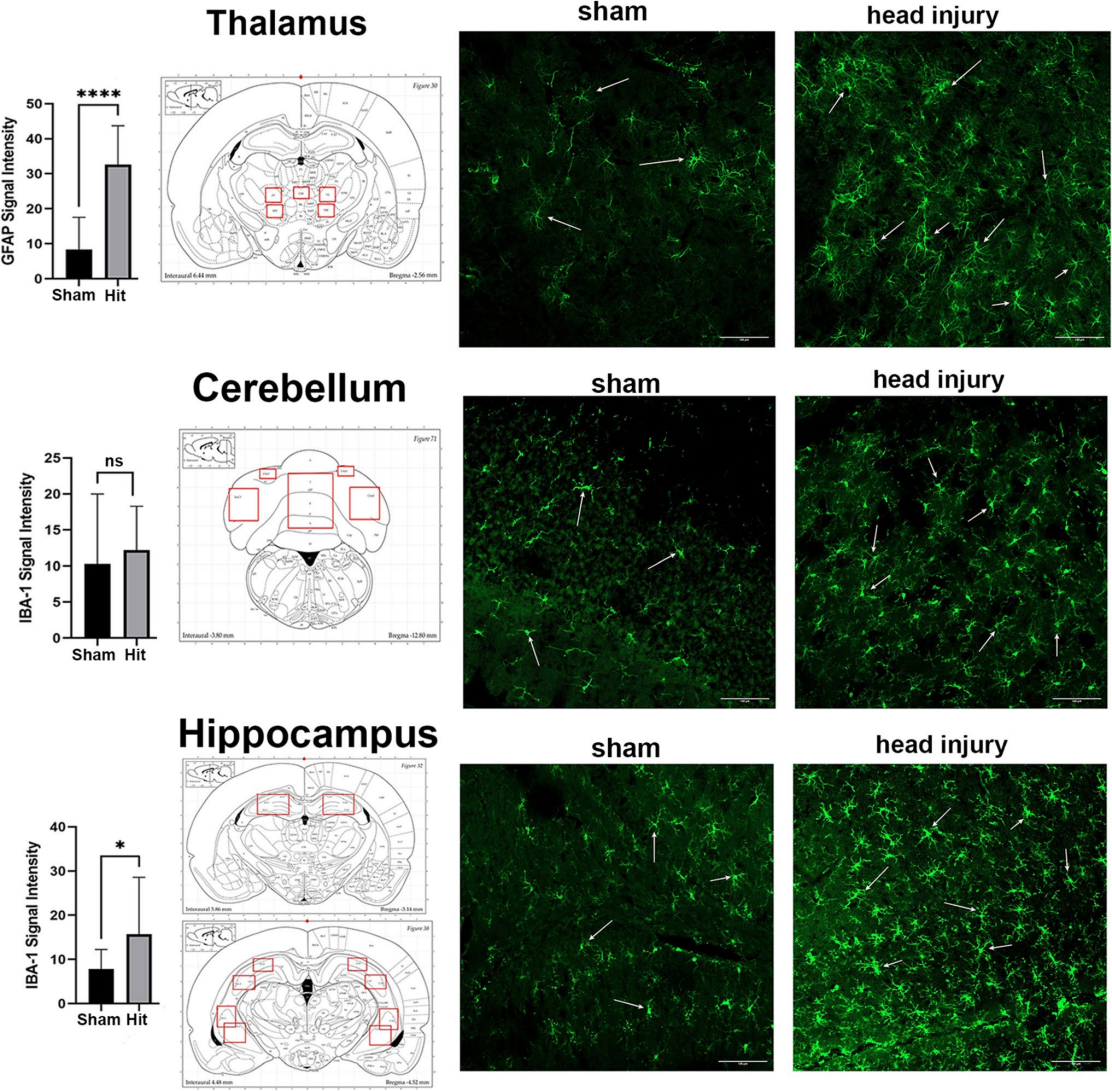
Gliosis histochemistry. The green, fluorescent images mark the presence of GFAP or Iba-1 for each of the experimental conditions. The approximate location of the images is shown in the red highlighted areas overlayed onto a rat atlas shown to the left. The signal intensities (mean ± SD) for GFAP in Sham (*n =* 5) and Head Injury (*n =* 4) and Iba1 Sham (*n =* 5) and Head Injury (*n =* 4) localized to the cerebellum, hippocampus, and thalamus are shown to the left. * P <0.05; **** P <0.0001.

Figure 6 shows the analysis for astrogliosis (GFAP) and microgliosis (Iba1) in different brain regions and examples of fluorescent micrographs for each. The areas sampled for gliosis in each brain region are delineated in the red boxes on the 2D sections taken from the Paxinos rat atlas [28]. The staining from the thalamus included the ventromedial, ventrolateral, and central medial nuclei. There was a significant increase (*P<*0.0001) in GFAP staining for head-impacted rats as compared to sham in the thalamus. The white arrows point to activated astrocytes. The staining for Iba1 from the cerebellum included Crus 1 & 2 and lobules 7,8, and 9 were not significantly different. The dentate gyrus, CA1, and CA3 regions were sampled from the hippocampus for Iba1 staining. Head-impacted rats showed significantly higher staining than shams (*P<*0.05). The white arrows in the florescent micrographs show activated microglia.

## Discussion

To make these preclinical studies more relevant to the human experience, repetitive mild head impacts were delivered during the dark period of the circadian cycle when rats are active, and then when female rats were awake without the confound of anesthesia. All studies were done on female rats knowing the recent multicenter studies on sex differences and the higher risk for women than men for post-concussive symptoms (PCS) impacting mental health [29, 30]. There was no neuroradiological evidence of skull damage brain contusion or noticeable deficits in motor behavior after three impacts. All of these findings attest to the mild nature of the head injury. With non-invasive multimodal MRI, we examined the brain for edema, BBB permeability, and functional connectivity. Postmortem histology ca. three weeks post-head impact confirmed areas of gliosis. These findings are discussed with respect to previous imaging studies on mild head injury and the relevance of this animal model to mild TBI in humans.

### Behavior with Mild Head Injury

Measures of learning and memory assessed with NOR and motor activity and anxiety assessed in the Open Field test were unremarkable and not significantly different between sham and head-impacted rats. This is not unexpected with mild head impacts in rodents. Anesthetized rats subjected to a single mild head impact [31, 32] or anesthetized mice with five mild head impacts separated by 24 hrs each [33] present with minor deficits in balance and motor coordination that recover within days. Ren *et al*. reported that anesthetized mice with a single mild impact present with no changes in cognitive function but do show a decrease in motor function on the rotarod that persists for up to 24 days [34]. Mice subjected to two mild repetitive head impacts while *fully awake* and screened for a battery of neurobehavioral tests show complete recovery within hours. [12]. Similarly, rmTBI in fully awake rats as reported in this study has no short-term effect on behavior. However, Xu and colleagues reported anesthetized male mice impacted daily for five consecutive days present with deficits in spatial memory in the Morris Water Maze when tested 16 weeks post injury [14]. Whether early insult in our study has long-term consequences on behavior with aging remains to be seen.

### Diffusion-Weighted Imaging

Edema plays a major role in the neuropathology of head injuries [35, 36]. Vasogenic edema results from damage to the blood-brain barrier (BBB), leading to the immediate movement of fluid into the brain's extracellular space. An increase in apparent diffusion coefficient (ADC), which measures water mobility, serves as an indicator of this volume change [36]. This increase in ADC is typically associated with a decrease in fractional anisotropy (FA). In cases of moderate to severe head injury, cytotoxic edema occurs, marked by cellular swelling due to disrupted osmolarity regulation across the plasma membrane. This type of edema generally shows a decrease in ADC and an increase in FA [37].

In a previous study involving anesthetized males during the light phase of the light-dark cycle, we found that a single mild impact, which showed no neuroradiological evidence of brain damage, led to a temporary increase in vasogenic edema in the thalamus, basal ganglia, and cerebellum, as indicated by a rise in ADC [16]. This increase in extracellular fluid volume peaked at 6 hours and returned to baseline by 24 hours. In the current study, awake female rats were subjected to three mild head impacts over three days and imaged for changes in ADC and FA within a few hours of the last impact. We expected that the severity of vasogenic edema in these rats would be greater than with a single impact, as indicated by an increase in ADC and a decrease in FA. Surprisingly, this was not the case. The hippocampus showed no differences in ADC and FA, while the cerebellum showed a decrease in FA and the thalamus a decrease in ADC. Most notable in the head-impacted rats were the significant changes in ADC and FA values in the basal ganglia that are suggestive of cytotoxic edema i.e., a decrease in ADC and an increase in FA.

### Blood Brain Barrier Permeability

Using dynamic contrast enhanced (DCE) imaging, Weissberg and colleagues studied the risk of BBB disruption with subconcussive injury in amateur football players as compared to control athletes in a non-contact sport [38]. Despite no previous evidence of concussion, 40% of the football players and 8.3% of the control athletes showed increased BBB permeability in gray and white matter localized to the cerebral cortex. DCE has also been used to study patients with post-concussive syndrome (PCS) following mild TBI [39] Months after insult these patients present with disruption in BBB permeability in the cerebral cortex, cerebellar white matter, and brainstem. It should be noted, that the extent of BBB disruption in patients evaluated with DCE is not limited to the area of impact or focal lesion but can extend to uninjured brain tissue distant from the site of injury [40].

There are several studies on BBB permeability following single and rmTBI in rodent models of closed-head impacts with no evidence of structural brain damage. Kane *et al*. reported no change in edema or BBB permeability following multiple head impacts in mice based on the wet/dry method to assess brain water content and histology for IgG, respectively [33]. Similarly, Ren *et al.* reported no edema or appreciable change in BBB permeability with Evans Blue extravasation in anesthetized mice after a single head impact [34]. Only 50% of *fully awake mice* subjected to mild head impacts present with a change in BBB permeability as measured by Evans blue extravasation and then only at the site of impact [12]. The change in BBB at the site of impact is transient and lasts for only three days. Logsdon *et al*. reported two mild blast injuries, only minutes apart, caused an immediate increase in BBB permeability across much of the brain [41]. In this model the change in BBB permeability is biphasic, disappearing within 24 hours but reappearing several days later. The late phase response is region-specific, localized primary to the frontal cortex, thalamus, medulla, and hippocampus. A reduction in claudin-5, a tight junction protein in endothelial cells, is thought to be responsible for the delayed changes in BBB permeability. A study from our laboratory using QUTE-CE imaging reported anesthetized, male rats subjected to three mild head impacts as described here present with exacerbated BBB permeability within 1–2 hrs after each head impact [19]. The changes are global affecting much of the brain. In the present study using QUTE-CE imaging, female rats exposed to mild repetitive head impacts during the dark period of the L-D cycle and while fully awake, show only minor changes in BBB permeability. These modest changes were localized to the cortex and cerebellum. These same areas were at risk as noted above for patients with PCS [39]. Interestingly, Hubbard *et al*. reported mild blast-induce head injury showed protein measures of BBB tight junction integrity that were reduced in males but not in females as compared to controls [42].

### Functional Connectivity

Patients studied within the first seven days of a mild TBI and presenting with PCS show a reduction in functional connectivity in the sensorimotor and central executive networks as compared to healthy volunteers [43]. Patients with mild TBI and PCS also show decreases in connectivity to the thalamus [44] and a decrease in the symmetry of connectivity between left and right thalamic nuclei [45]. The connectivity between the motor-striatal-thalamic network is reduced in mild TBI while the frontoparietal network is increased [46]. Pinky *et al*. reported a decrease in functional connectivity in the cerebellum and basal ganglia in sports-related concussions in 12–18-year-olds [47]. The cerebellar gray matter also showed increases in magnetic susceptibility thought to be due to acute inflammation and accumulation of iron. Most recently Fitzgerald and colleagues ran longitudinal studies on young athletes competing in American football collecting functional connectivity prior to, during, and following the season [48]. Self-patterns of functional connectivity declined during the playing season but recovered after the season ended. They proposed that exposure to multiple head acceleration events may cause changes in brain neurobiology that are similar to concussion but in the absence of any symptomatology.

In an earlier study, using the three-hit model described here but performed on male rats under anesthesia and during the light phase of the L-D cycle, we reported an increase in connectivity in response to a single head impact but a decrease with multiple impacts two months post insult [17]. In the present study, there is a significant decrease in connectivity in the cerebral cortex, hippocampus, cerebellum, and thalamus. In a recent study, Sakthivel and coworkers reported impairment in connectivity in male and female mice exposed to two mild head impacts separated by 24 h each [15]. There was a significant decrease in global and local efficiency particularly in the thalamus.

### Histopathology

Histology sections were collected from the thalamus, hippocampus, and cerebellum, areas showing neuroradiological changes with head injury. Immunostaining for gliosis as determined by activated astrocytes and microglia showed putative inflammation in the thalamus and hippocampus. The changes in thalamic and hippocampal gray matter microarchitecture as identified with diffusion-weighted imaging and the decrease in functional connectivity may be imaging biomarkers of the underlying cellular pathology.

### Data Interpretation

What constitutes a “mild” head injury? The Glasgow Coma Scale of 13–15 defines head injury as “mild” based on measures of motor behavior, verbal responses, and eye-opening, with loss of consciousness and short hospitalization [49]. With the advent of CT and MRI, physical damage to the brain could be confirmed, adjusting the classification to “mild with complication” [50]. Organized sports and head trauma on the battlefield added another dimension to the characterization of “mild.” The Centers for Disease Control and Prevention, WHO, and American Congress of Rehabilitation Medicine for diagnosing mild head injuries include self-reports of transient confusion, disorientation, impaired consciousness, or dysfunction in memory around the time of the injury - importantly there should be no structural damage as determined with CT or MRI [2, 3]. This study in female rats used multiple head impacts, separated over time, all of which were “mild” confirmed by the absence of any structural brain damage by MRI (see Fig. 1). Our previous publications on repetitive mild head injury in the anesthetized male rat during the light phase of the L-D cycle met these criteria [17, 19, 24]. However, when comparing those studies in males, that ignored the importance of circadian biology and the confound of anesthesia, with data collected from females studied without anesthesia and during the dark phase of the L-D cycle there are significant differences. The risk for disruption in the BBB was much less in this study as compared to our previous work in males [19]. The robust increase in ADC values indicative of vasogenic edema noted in anesthetized males was absent in this study on unanesthetized females [16]. Instead, there was a decrease in ADC and increased FA values in the basal ganglia which would suggest the presence of cytotoxic edema that might be expected with repetitive head injury. These differences may be due to sex, the presence of anesthesia, or the influence of circadian biology. The importance of circadian biology on brain function cannot be ignored particularly in light of the recent work on the glymphatic system, perivascular clearance, and BBB permeability during the resting/sleep phase of the L-D cycle [51–53]. What was most consistent between the two protocols was the disruption in functional connectivity, a finding that readily translates to the human condition, and the presence of gliosis indicative of neuroinflammation.

The absence of any significant changes in behavior is not evidence that there were no changes in behavior. While novel object recognition and open field are two commonly used behavioral assays to assess motor behavior, cognition, and anxiety, interrogation with other behavioral assays may have parsed out subtle differences between control and head-impacted rats.

It is interesting that the area immediately under the approximate site of impact does not show localized neuropathology but only the edema in the overlaying skin. This is a closed skull momentum exchange model with diffuse neuropathology. The most likely explanation for the extended neuropathology is coup counter-coup and shearing of white matte tracts in distant locations resulting in localized changes in BBB permeability and neuroinflammation.

## Summary

A recent review by Cox *et al.* questioned the effectiveness of preclinical models in advancing the development of new treatments for head injuries [54], a perspective echoed by others to explain the numerous failed clinical trials for traumatic brain injury (TBI) [55, 56]. Consequently, we chose to move away from traditional TBI models that typically result in brain damage and subsequent cognitive and motor dysfunction. Instead, we focused on mild head injuries commonly encountered in organized sports, military combat, and everyday accidents among both young and elderly individuals. A key aspect of this model is the absence of detectable brain damage, as confirmed by neuroradiology. The only indication of injury is a superficial"bump on the head"due to edema on the skin over the skull, as illustrated in Fig. 1. This model has been discussed in previous studies on mild head injury. [12, 31, 33, 34, 57], does not effectively alter behavior discounting this measure as an endpoint when interpreting disease progression and drug efficacy using rodents. To make our model more relevant to the human experience rats were head impact while fully awake eliminating the confound of anesthesia, and during the dark phase of their L-D cycle when they are most active. Magnetic resonance imaging for changes in indices of anisotropy using DWI, BBB permeability using blood contrast enhanced techniques, and functional connectivity are readily performed in the clinic aiding in the translation of data from rodents to humans by using the same techniques. Previous studies from our lab using imaging with mild impacts directed to the forehead have identified the thalamus, cerebellum, hippocampus, basal ganglia, and midbrain dopaminergic system as vulnerable areas [16, 17, 19, 24, 58]. In this study, designed to better reflect the human experience the cerebellum, basal ganglia, and thalamus were most sensitive to repeated insult. Indeed, the thalamus was highlighted as a region sensitive to mild TBI as noted here using DWI, rsFC, and histology. The sensitivity of the thalamus was also noted in many clinical studies cited above for rsFC.

## Supplementary Information

Below is the link to the electronic supplementary material.

Supplementary file1 (PDF 138 KB)

Supplementary file2 (XLSX 20 KB)

Supplementary file3 (XLSX 21 KB)

Supplementary file4 (XLSX 15 KB)

## Data Availability

The datasets generated during and/or analyzed during the current study are available from the corresponding author upon reasonable request.
