# Supplementary figures and images for: Multimodal Magnetic Resonance Imaging with Mild Repetitive Head Injury in Awake Rats: Modeling the Human Experience and Clinical Condition
Source: Neurosci Bull. 2025 Jun 29;41(9):1603–16. doi: 10.1007/s12264-025-01438-9 (PMC12433400; doi:10.1007/s12264-025-01438-9)

## Supplementary Figure S1

**Fig. S1** Experimental Time Course

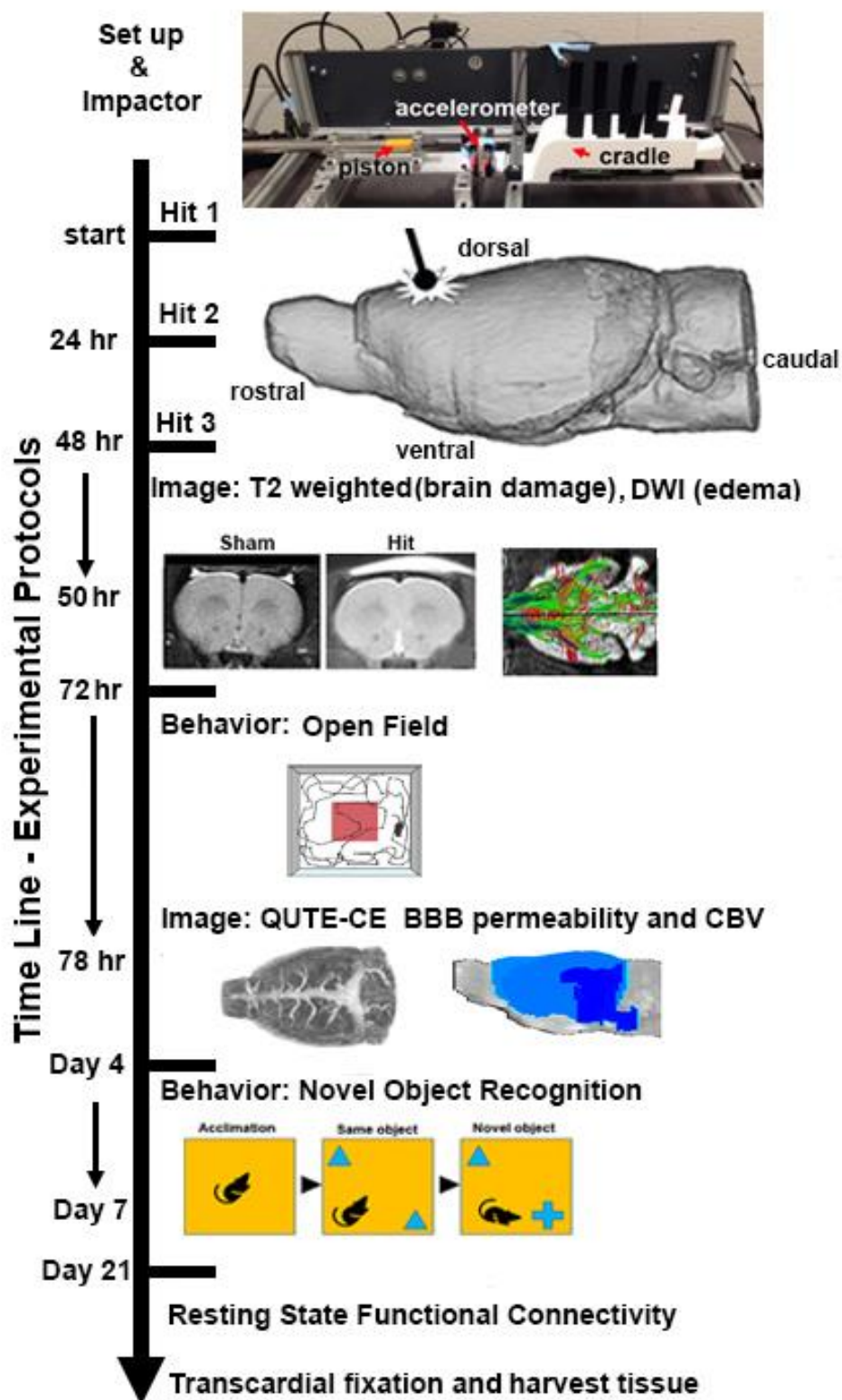

Supplement: Supplementary file 1 — Supplementary file1 (PDF 138 KB) [file 12264_2025_1438_MOESM1_ESM.pdf]
